# Impact of nitrogen-fixation bacteria on nitrogen-fixation efficiency of Bambara groundnut [*Vigna subterranea (L) Verdc*] genotypes

**DOI:** 10.3389/fmicb.2023.1187250

**Published:** 2023-09-26

**Authors:** Tope Daniel Bitire, Michael Abberton, Esther Oluwabukunola Tella, Alex Edemodu, Olaniyi Oyatomi, Olubukola Oluranti Babalola

**Affiliations:** ^1^Food Security and Safety Focus Area, Faculty of Natural and Agricultural Sciences, North-West University, Mmabatho, South Africa; ^2^Genetic Resources Center, International Institute of Tropical Agriculture (IITA), Ibadan, Nigeria; ^3^Department of Microbiology, Faculty of Natural and Applied Science, Lead City University, Ibadan, Nigeria; ^4^Yam Breeding Unit, International Institute of Tropical Agriculture (IITA), Ibadan, Nigeria

**Keywords:** bacteria strain, inoculation, nitrogen fixation, nodules, underutilized legumes

## Abstract

Nitrogen fixation by bacteria is essential for sustaining the growth, development, and yield of legumes. Pot experiments were carried out at the International Institute of Tropical Agriculture (IITA) in the glasshouse between August to December 2018/2019 cropping season in Ibadan, Nigeria. Field studies were also performed in two different agroecological zones, “Ibadan and Ikenne” between August and December of 2019/2020 cropping season. The studies were set up to determine the potential of nitrogen-fixation bacteria strain inoculation on the nitrogen-fixation potential of 10 Bambara groundnut (BGN) genotypes, namely, TVSu-378, TVSu-506, TVSu-787, TVSu-1,606, TVSu-1,698, TVSu-1739, TVSu-710, TVSu-365, TVSu-475, and TVSu-305. The strains were inoculated as a broth to seedlings of each BGN genotype in the pot experiment. While six seeds from each BGN genotype were coated with each of the following nitrogen-fixation bacteria (*Bradyrhizobium japonicum* strains), FA3, USDA110, IRJ2180A, and RACA6, nitrogen fertilizer (urea, 20 kg/ha) was applied as a check to the nitrogen-fixation bacteria to seedlings of BGN genotypes 2 weeks after planting in both glasshouses and fields. Uninoculated plants served as controls (zero inoculation and zero fertilization). The field experiments were arranged in Randomized Complete Block Design (RCBD), while the glasshouse experiments were arranged in Complete Randomized Design (CRD) in triplicate. The result gotten showed that higher nodule numbers and weight were recorded in TVSu-1739 and TVSu-475 in both locations and seasons compared to other genotypes; the highest nitrogen fixed values were recorded among BGN genotypes TVSu-1739, TVSu-1,698, TVSu-787, TVSu-365, TVSu-305, TVSu-710, and TVSu-1,606, with a range of (62–67 kg ha^−1^), and were mostly enhanced by RACA6 and USDA110 strains compared to other strains that were used.

## 1. Introduction

The most available gas constituting a major part of the atmosphere is nitrogen. However, only the reactive types of nitrogen like oxidized or reduced nitrogen types can be assimilated by plants (Burén and Rubio, 2018). Urea, amino acids (proteins), nucleic acids (DNA and RNA), adenosine triphosphate (ATP), and nicotinamide adenine dinucleotide (NAD) are components of nitrogen in all living cells (Burén and Rubio, 2018). Nitrogen gas (N_2_) conversion to the biologically available form of nitrogen (NH_3_) could be performed either by the industrial Haber-Bosch process or via biological nitrogen fixation by certain bacteria. Different types of plant-beneficial microorganisms live in the soil and usually interact with plants and other types of microorganisms (Igiehon and Babalola, 2018). Interaction between rhizobia and legume type is usually determined by nodulation from rhizobia and the structure of Nod factor in the host (Oldroyd et al., 2011). The process between the rhizobia and the legume is usually initiated by the host plant, which exudes from the root flavonoid molecules that are recognized by their rhizobia partners (Ferguson et al., 2010; Sprent et al., 2017). These flavonoids attract the bacteria to the roots and help them to produce nod factor molecules in the host plants, leading to the formation of root organs called nodules (Ferguson et al., 2010). These nodules, therefore, provide an environment conducive to biological nitrogen fixation, which the rhizobia release for their host plant in exchange for carbohydrates (Ferguson et al., 2019). The prolonged use of agrochemicals has been proven to affect soil fertility and structure, water holding capacity, and diversity of beneficial microorganisms (Enagbonma and Babalola, 2019). *Rhizobium* was the first discovered genus of bacteria to fix nitrogen, which is why this name has been used often for the nitrogen-fixation bacteria of legumes. Approximately 112 species are accommodated by *rhizobium,* which is the largest genus of rhizobia (Lindström and Mousavi, 2020). The *nod*, *nif*, and *fix* genes usually control the symbiotic nitrogen fixation in rhizobia. These sets can be transferred horizontally in high frequencies within the species of a bacterial genus and infrequently between genera (Remigi et al., 2016). BGN is a leguminous crop that is under-researched and underutilized in Africa. Usually, BGN can fix approximately 4 to 200 kg N ha^−1^ through a symbiotic relationship with soil bacteria called ‘rhizobia’ (Puozaa et al., 2017). There is little information on the biodiversity of rhizobia that nodulate BGN in African soils; nevertheless, some research studies have shown that BGN can be nodulated by species of the genus *Bradyrhizobium* (Grönemeyer et al., 2014). As rhizobia species nodulating most legumes can vary, inoculation with stress-tolerant strains of *rhizobium* may enhance the nodulation and nitrogen fixation ability of BGN under stress conditions. There has been very little research carried out on nitrogen fixation in BGN (Mohale et al., 2014). Bitire et al. (2022) recorded a substantial increase in the growth and yield of Bambara groundnut when inoculated with bacteria strains compared to the yield recorded when chemical fertilizers were applied to the legume. Hassen et al. (2023) reported an increase in the nodule number and plant fresh and dry weight in Bambara groundnut inoculated with rhizobia strains. Based on a study conducted by Yakubu et al. (2010), BGN has a high nitrogen fixing potential, provided that an effective and appropriate *rhizobium* strain is used as an inoculant and that the plant nutrient requirements other than nitrogen are readily available in the soil. Adegboyega et al. (2021) reported that the amount of N fixed (kg ha^−1^) in the shoot of wing bean varies among accessions. Zoundji et al. (2022) concluded that the inoculation of Bambara groundnut with rhizobia improved nodulation and dry biomass yield and enhanced nitrogen uptake. Therefore, the aim of this experiment is to determine the potential of nitrogen-fixation bacteria strains on the nitrogen fixation of different BGN accessions that differ in genetic composition.

## 2. Materials and methods

### 2.1. Pot experiments

Pot experiments were carried out in the glasshouse at the International Institute of Tropical Agriculture in Ibadan [Latitude (Lat) 7° 22′ 30 N and Longitude (long) 3° 45′ 54 E], Nigeria Planting in August and harvesting in December, with a temperature of 33° C, relative humidity of 48%, and light intensity of 40%. The soil samples (sandy loam) were sieved through a 3 mm sieve and sterilized at 121°C for 1 h using the autoclave before planting (Mendoza-Suárez et al., 2021). The sterile and non-sterile soil were filled separately in 10 kg pots and were carefully arranged in the glasshouse in a complete randomized design (CRD) in three replicates. The seeds of BGN genotypes were surface sterilized, two seeds were sown in a 10 kg pot containing sterile and non-sterile soil in the glasshouse, and plastic pegs showing BGN genotypes and treatments were placed in the pots for easy identification. The temperature, relative humidity and light intensity are shown above. The experiment is a 10×6 factorial arrangement. Ten Bambara groundnut genotypes, namely, TVSu-378, TVSu-506, TVSu-787, TVSu-1,606, TVSu-1,698, TVSu-1739, TVSu-710, TVSu-365, TVSu-475, and TVSu-305, were randomly selected at the International Institute of Tropical Agriculture (IITA), Gene bank Ibadan, Nigeria. A total of 10 days after emergence, seedlings of the BGN genotypes were inoculated with 3 mL of the broth form of the (nitrogen-fixation bacterial) *Bradyrhizobium japonicum* strains: FA3, RACA6, USDA110, and IRJ2180A containing 2.8×10^7^, 7.2×10^6^, 4.3×10^7^, and 1.4x10^7^cfu/ml, respectively. N fertilizer was also applied at the rate of 20 kg/ha (recommended rate of N fertilizer for Bambara groundnut cultivation) in the form of urea (46%) to the seedlings of BGN genotypes that were not inoculated, and an un-inoculated control and maize was planted as a reference crop, also in triplicate. The choice and the types of strains used in this study were determined by their availability at the Soil Microbiology Laboratory of the (IITA), Ibadan, Nigeria. The hosts of the bacteria were mainly soybean and BGN. Experimental site and soil sterility did not influence the results and were, therefore, grouped together. Nitrogen-free nutrient solution and sterile water were added to each pot at regular intervals, twice a week (Davies and Owen, 1951). At 50% flowering, plant samples were uprooted (destructive sampling), oven-dried at 65°C for 2 days, and carefully ground using the grinding machine. The dried samples were used to determine the total N available by the Macro Kjeldahl oxidation method, which entails digestion and distillation. The nitrogen content of the inoculated accessions was determined using the N difference method, as described by Yakubu et al. (2010).

% N derived from atmosphere (Ndfa) was calculated using the following formula:


$$X=\frac{D\times N}{V}$$

Y=L−R

$$K=\frac{L-R\times 100}{L}$$

Where

X = Total N in plants

D = Dry matter weight

N = %N in plants

V = 100

Y = N fixed

L = Total N in legume

R = Total N in reference crop (maize)

K = % (Ndfa)

### 2.2. Field experiments

The 10 BGN genotypes used in the pot experiments were also used in the field from August to December 2019/2020 in two geographical locations and seasons: Ibadan (7^o^ 38’N, 3^o^ 89′E) and Ikenne (6^o^ 86’N, 3^o^ 71′E), Nigeria. The soil (sandy loam) in both locations was carefully pulverized using a tractor-driven plow, and 2 m ridges were made with intra spacing of 1 m between ridges. Six seeds from each BGN genotype were coated with Arabic gum and nitrogen-fixation bacteria FA3, USDA110, IRJ2180A, and RACA6 separately and allowed to dry before being planted in both geographical locations and seasons; N fertilizer 20 kg/ha (recommended rate of N fertilizer for Bambara groundnut cultivation) in the form of urea (46%) was applied to seedlings that were not coated 2 weeks after emergence. The experiments were arranged in a randomized complete block design (RCBD) in three replicates (block). Plastic pegs containing the BGN genotypes and treatments administered were placed at the edge of each ridge for easy identification. Seeds that were not coated served as controls and maize was planted as a reference crop in triplicate. Weeding was done manually at regular intervals using a hoe. At 50% flowering, plant samples were collected from the fields (Ibadan and Ikenne) in both seasons, 2019 and 2020, respectively, at the same time. The samples obtained were taken to the Soil Microbiology Laboratory unit (IITA). Plant roots were carefully washed to recover the nodules. The nodules were counted and weighed on a Mettler balance to determine the fresh weight. The fresh weight of the shoots was also recorded and samples were oven-dried at 65°C for 2 days. The dried samples were carefully ground and used to determine the estimation of N fixation on the field using the Ureide method.

The relative abundance of ureide in stem extract can be calculated as

$\mathrm{Relative}\;\mathrm{Ureide}\;N=\frac{K\times a}{La+b}$

Where N = N%.

K = 400.

a = Molar concentration of ureides.

b = molar concentration of nitrates.

L = 4 (Herridge and Peoples, 1990).

### 2.3. Ureide determination

#### 2.3.1. Extraction

Precisely 0.5 g dried and ground stems were transferred to a 100 mL beaker, and 25 mL distilled water was added to each subsample and boiled for 1–2 min. The samples were filtered into a 50 mL volumetric flask using 15 cm filter paper and were made up to 50 mL with distilled water. Eluent was kept and stored in vials in a freezer (−18°C) until the analysis of nitrogen solutes (Shamseldin, 2013).

#### 2.3.2. Reagent preparation

Precisely 2 g sodium hydroxide (NaOH) was added to a 100 mL beaker with the further addition of 6.5 mL concentrated hydrogen Chloride (HCl). Furthermore, 0.33 g phenylhydrazine and 1.67 g of Potassium ferricyanide were added to 100 L distilled water. Furthermore, 400 mL concentrated HCl was decanted from the bottle and 39.53 mg allantoin was added to 250 mL distilled water (Shamseldin, 2013).

#### 2.3.3. Procedure

Precisely 2.5 mL of each of the five concentrations was added to duplicate test tubes with 0.5 mL 0.5 Normal sodium hydroxide. The tubes were incubated in a boiling water bath for 10 min. Then a mixture of 1.0 mL 0.65 Normal HCl and phenylhydrazine inside the tubes was placed in boiling water for exactly 2 min. The tubes were later incubated in ice for 15 min, after which, 2.5 mL of HCl/KFeCn was added and mixed thoroughly and allowed to stay for 10 min for the development of color and accurate results. The absorbance of the samples was measured at 525 nm using a spectrophotometer (Peoples et al., 1989).

### 2.4. Nitrate determination

#### 2.4.1. Reagent preparation

Precisely 5 g salicylic acid was added to 100 mL H_2_SO_4_ in a beaker. Then, 40 g NaOH was added to 500 mL distilled water, and 632 mg KNO_3_ was added to 250 mL distilled water.

#### 2.4.2. Procedure

Precisely 0.05 mL of each concentration was added using a pipette into duplicates test tubes of each of the concentration was added using a pipette into duplicate test tubes and 0.02 mL of salicylic acid was added using a pipette into the tubes and was allowed to mix and leave on the bench for 20 min. The solution was allowed to clear and left on the bench for 10 min and was later read at an OD (optical density) of 410 nm on a spectrophotometer.

**Figure 1:**
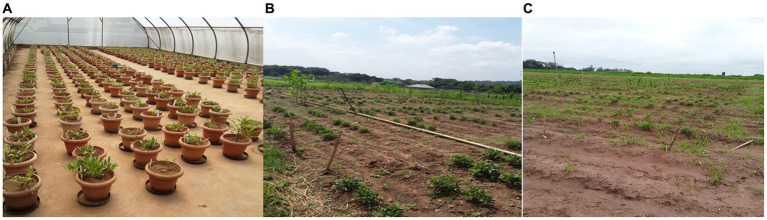
**(A)** BGN genotypes in a sterile and non-sterile soil. **(B)** BGN genotypes inoculated in Ibadan **(C)** BGN genotypes inoculated in Ikenne.

### 2.5. Data analysis

The data collected were subjected to four-way ANOVA (Analysis of Variance): pot (accessions, strains, soil status, and season), field (accessions, strains, locations, and seasons) statistical analysis system (SAS) package 9.4, and means were separated using the Duncan Multiple Range Test (Dmrt) at *p* ≤ 0.05 (Zatybekov et al., 2017).

### 2.6. Characterization of the soil used in the studies

The pH values of the soils used in the glasshouse in both seasons were neutral, the pH values of the soil in Ikenne were acidic in nature in both seasons, while in Ibadan, the pH values were slightly acidic. The organic carbon percentage was determined using the Walkley black chromic acid wet oxidation methods, and it ranged from 0.25 to 0.40 in both the glasshouse and in the field, which shows the soil used was normal. The P in the soil was determined using the wet digestion method, the phosphorus in the soil used in the glasshouse was low, while a higher percentage of P was recorded in the soil used in the field in both locations and seasons, ranging from 15.35–52.84 ppm. Figure 1 shows the cultivation of inoculated BGN in a sterile and non-sterile soil, and in soils in both locations, Ibadan and Ikenne. Available nitrogen in the soil was determined using the Kjeldahl methods, and the results obtained showed that the N in the soil in the glasshouse was low in both seasons and high in the field experiment in both locations and seasons. The K in the soil was extracted using ammonium acetates and analyzed using the atomic absorption photometer. The percentage of K present in the soil used in both the glasshouse and in the field in both seasons shows availability in moderate quantities. The Ca in the soil used in the glasshouse was extremely high, while the Ca in the field in Ibadan was considered normal (Table 1). The Mg present in the soil used for the glasshouse experiments was high in both sterile and non-sterile soil in both seasons. Higher Mg values were obtained in Ikenne in both seasons, while lower quantities of Mg were obtained in Ibadan in both seasons. The particle size analysis was determined by the (hydrometer method). The soil textural class shows that the soil used both in the glasshouse and in the field was a loamy sand (Table 1). The Biological properties, which include AM fungi, were determined using the sucrose density gradients centrifugation methods, which involve the use of sucrose to isolate available mycorrhizal fungi in the soil. The available spore was determined using wet sieving and decanting methods the spore count in 100 g soil was achieved using the counting methods under a dissection microscope. The soil textural class shows that the soil used in the glasshouse and on the field in both locations and seasons shows that the soil was loamy sand.

**Table 1:** Physicochemical and biological properties of soil used for studies both in the glasshouse and field.

Experiment	Pot experiment				Field experiment			
Year	2018	2018	2019	2019	2019	2020	2019	2020
Soil status/ Location	Sterile soil	Non-sterile soil	Sterile soil	Non-sterile soil	Ikenne	Ikenne	Ibadan	Ibadan
pH (H_2_O)	7.43	7.39	7.1	6.81	4.46 ± 0.08	4.91 ± 0.09	6.84 ± 0.1	7.13 ± 0.11
N (%)	0.05	0.04	0.05	0.10	0.073 ± 0.02	0.121 ± 0.01	0.150 ± 0.013	0.119 ± 0.02
OC (%)	0.38	0.40	0.03	0.25	0.329 ± 0.07	0.297 ± 0.01	0.407 ± 0.10	0.336 ± 0.06
Bray P (ppm)	3.30	3.71	2.55	3.71	13.23 ± 4.26	22.46 ± 3.43	15.352 ± 3.74	52.84 ± 6.67
Sand (%)	81.00	83.00	77.0	81.00	76.00 ± 1.16	76.00 ± 1.16	83.00 ± 1.16	82.00 ± 0.0
Clay (%)	13.00	11.00	19.00	11.00	16.00 ± 1.16	20.00 ± 2.00	10.00 ± 0.00	8.00 ± 0.00
Silt (%)	6.00	6.00	4.00	8.00	6.00 ± 1.15	3.00 ± 1.15	7.00 ± 1.15	10.00 ± 0.0
Ca (Cmol/kg)	6.74	5.92	8.53	8.63	3.530 ± 2.50	1.505 ± 0.24	1.216 ± 0.10	1.101 ± 0.30
Mg (Cmol/kg)	6.74	5.92	0.20	0.20	0.80 ± 0.37	0.404 ± 0.02	0.075 ± 0.01	0.158 ± 0.05
K (Cmol/kg)	0.24	0.22	0.27	0.29	0.560 ± 0.24	0.242 ± 0.06	0.137 ± 0.02	0.201 ± 0.02
Na (Cmol/kg)	0.06	0.06	0.08	0.08	0.076 ± 0.01	0.082 ± 0.00	0.055 ± 0.02	0.057 ± 0.01
ECEC (Cmol/kg)	13.79	12.12	9.08	9.21	4.96 ± 1.56	2.23 ± 0.57	1.29 ± 0.56	1.47 ± 0.48
Zn (ppm)	3.24	2.66	9.91	9.39	1.964 ± 1.67	1.196 ± 0.19	1.19 ± 0.10	0.66 ± 0.04
Cu (ppm)	0.95	0.49	0.64	0.70	1.167 ± 0.62	2.045 ± 0.19	0.86 ± 0.11	0.97 ± 0.22
Mn (ppm)	266.0	286.2	214.7	222.1	12.15 ± 10.5	116.7 ± 3.31	244.20 ± 18.4	383.6 ± 33.4
Fe (ppm)	17.19	17.19	85.54	94.01	25.58 ± 7.47	88.29 ± 4.08	133.33 ± 3.33	114.4 ± 3.85
AM fungi	-	159	-	145	106	117	135	152
*Acaulospora*	-	211	-	200	192	185	202	208
*Enthrophospora*	-	30	-	36	25	28	34	32
Spore/100gdwt	-	401	-	406	380	392	412	432
Soil textural class	Loamy sand	Loamy sand	Loamy sand	Loamy sand	Loamy sand	Loamy sand	Loamy sand	Loamy sand

## 3. Results

### 3.1. Response of BGN genotypes to inoculation

**Figure 2:**
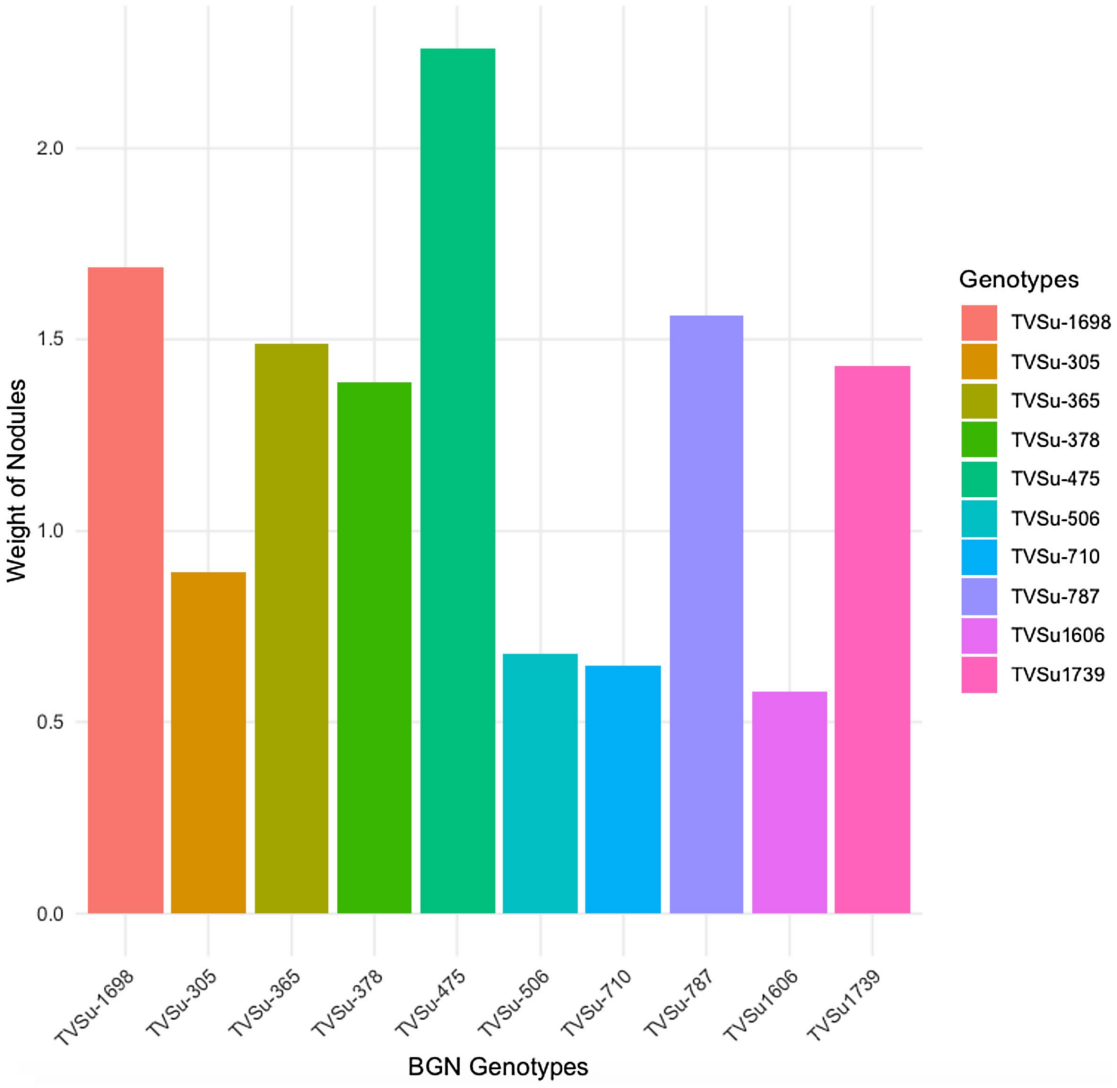
Effect of nitrogen-fixation bacteria strains on nodule weight of BGN genotypes in both locations and seasons.

The analysis of variance showed that significant differences were recorded among the inoculated BGN genotypes, nitrogen-fixation bacteria inoculation, and the interaction of seasons with locations in nodulation and nitrogen fixation recorded in both geographical locations and seasons (Table 2). There were no significant differences recorded in the interaction between genotypes and strains, genotypes and locations, and strains and locations, nor in the interactions among genotypes, strains, location, and season (Table 2). The inoculation of BGN genotypes with nitrogen-fixation bacteria strains revealed that higher nodule numbers were recorded in TVSu-1739, with a mean value of 12.48, but not significantly different from TVSu-475, with a mean value of 10.88, but are significantly different from other BGN accessions inoculated in this study in both geographical locations in 2020 (Figure 2). Moreover, no significant difference was recorded between TVSu-1,698 and TVSu-475 in the weights of nodules recorded, but they were significantly higher than other inoculated BGN genotypes in the study. There were also no significant differences observed among the inoculated strains in the number of nodules recorded, but the FA3 strain showed a greater significant difference than other strains in the nodule weight recorded. Nitrogen-fixation bacteria strains (FA3, USDA110, IRJ2180A, and RACA6) are significantly higher than the Nitrogen fertilizer applied and the uninoculated control in the nodule number recorded and nitrogen fixed in both seasons and locations (Table 3), which makes the inoculation of BGN genotypes with bacterial strains superior to the nitrogen fertilizer (urea) applied in both the pot and field studies.

**Table 2:** Analysis of variance showing the effect of nitrogen-fixation bacteria strains on nodulation and nitrogen fixation of BGN genotypes in both seasons (Ibadan and Ikenne).

Source	DF	Nodule number	Nodule weight (g)	Nitrogen fixed (kg/ha)
Genotypes	9	629.24**	19.55**	217.84*
strains	5	97.38^**^	3.59^ns^	15336.58**
season	1	204.68*	797.11**	5548.10**
location	1	22.63^ns^	25.07**	823.69**
Genotypes*strains	45	40.51^ns^	1.89^ns^	126.58^ns^
Genotypes*season	9	168.21**	18.14**	287.71**
Genotypes*location	9	72.31^ns^	3.42^ns^	151.19^ns^
strains*season	5	52.73^ns^	2.51**	348.06**
season*location	1	1393.73**	79.67**	6.19^ns^
strains*location	5	66.09^ns^	1.66^ns^	156.95^ns^
Genotype*strain*locat*season	131	32.38^ns^	1.94^ns^	111.66^ns^
Rep	2	47.83^ns^	29.57**	116.09^ns^

Key: DF-Degree of freedom, **p < 0.01 Significant, *p < 0.05 highly significant, ns-not significant.

**Table 3:** Effect of nitrogen-fixation bacteria strains on nodulation and nitrogen fixation of BGN genotypes in both seasons (Ibadan and Ikenne).

Strains	Nodule number	Nodule weight (g)	Nitrogen fixed (kg/ha)
FA3	7.80^a^	1.16^a^	69.95^b^
IRJ2180A	7.49^a^	1.17^a^	72.02^b^
RACA6	7.32^ab^	1.47^a^	75.95^a^
USDA110	6.92^ab^	1.26^a^	73.32^ab^
N	5.35^b^	1.06^a^	42.41^c^
Control	7.07^ab^	1.42^a^	44.07^c^

Means with the same letters are not significantly different at a 5% level of probability using the (DMRT).

**Figure 3:**
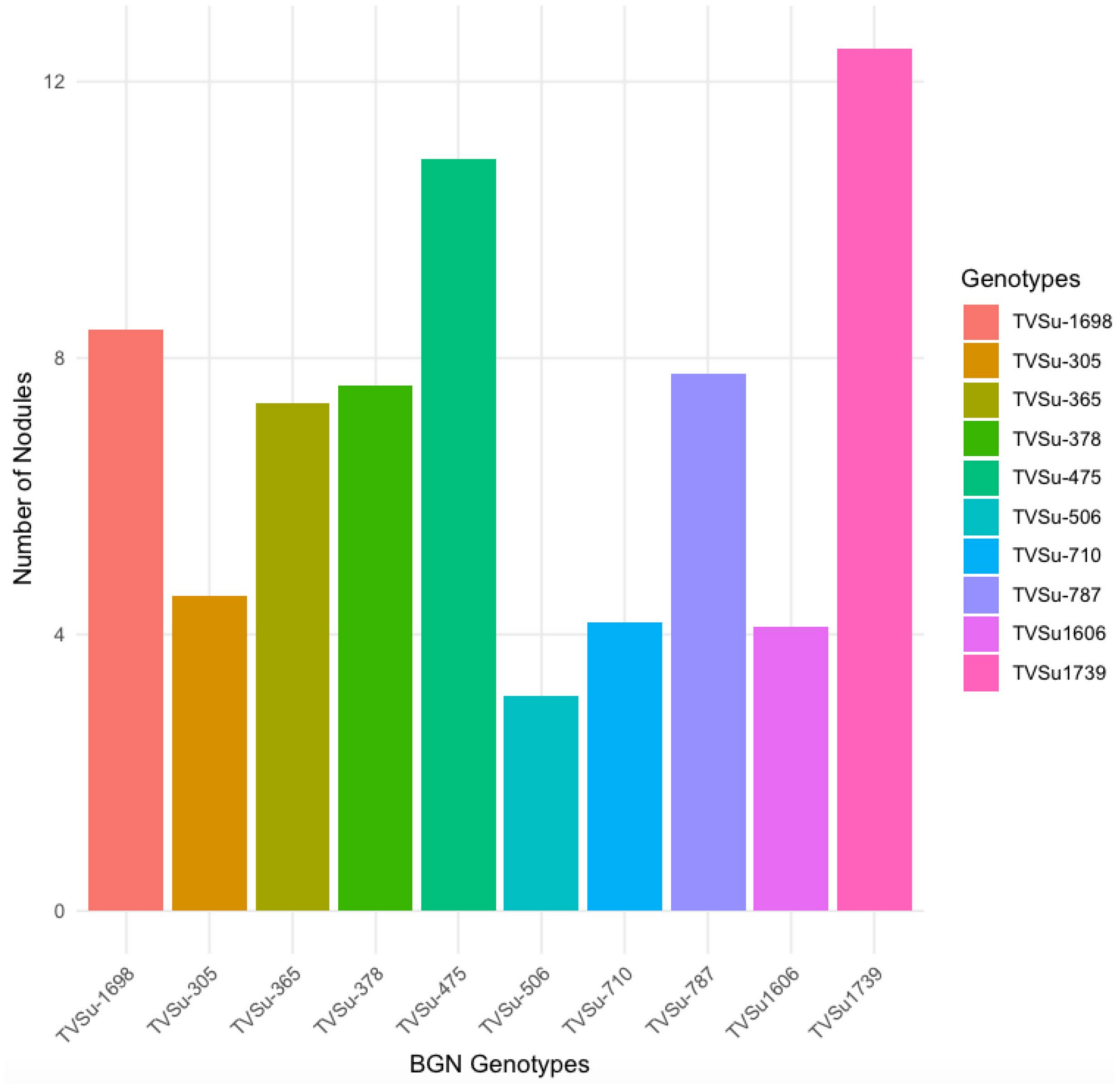
Effect of nitrogen-fixation bacteria strains on number of nodules of BGN genotypes in both locations and seasons.

**Figure 4:**
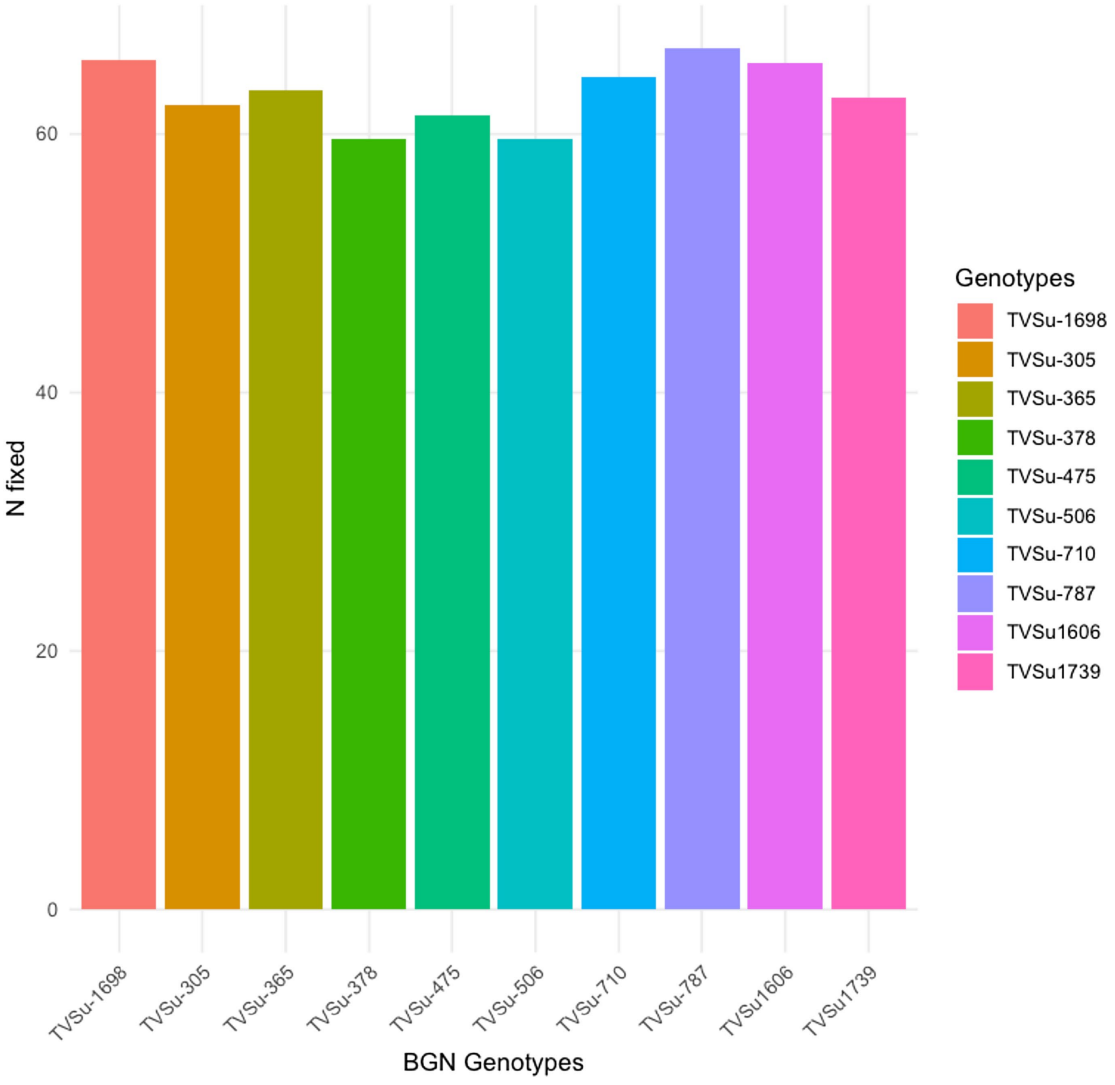
Effect of nitrogen-fixation bacteria strains on nitrogen fixation of BGN genotypes in both locations and seasons.

Moreover, RACA6 and USDA110 strains are not significantly different in the nitrogen fixed values but significantly different than other nitrogen-fixation bacteria, N fertilizer application (urea), and uninoculated controls in both locations and seasons (Table 3). No significant differences were recorded among the bacteria strains, nitrogen fertilizer, and uninoculated control in the nodule weight in both seasons and locations, which may be due to the level of indigenous rhizobia also nodulating the legume (Table 3). The results revealed that the inoculation of BGN genotypes with strains significantly enhanced nodulation and nitrogen fixation of legumes compared with the nitrogen fertilizer applied and the uninoculated control (Table 4). Interaction of TVSu-1,698 with FA3 strains enhanced the nodule number recorded compared to other genotypes with strains interaction (Table 4). The highest nodule weight was recorded in the interaction of TVSu-1,698 with control as a result of the indigenous rhizobia present in the soil used (Table 4). The highest nitrogen fixed was recorded in the interaction of TVSu-1,606 with RACA6 compared to other genotypes with strains interaction (Table 4). Furthermore, nitrogen-fixation bacteria inoculation also enhanced the nodule number and nitrogen derived from the atmosphere (Ndfa) of BGN genotypes inoculated in the glasshouse (Table 5). Significant differences were recorded among the genotypes in the nodule number and nitrogen derived from the atmosphere, but no significant differences were recorded in the nodule weight and nitrogen fixed among the Bambara groundnut genotypes (Table 5). Furthermore, significant differences were recorded among the strains in the nodule number, weight, and nitrogen fixed, but no significant differences were recorded in the amount of nitrogen derived from the atmosphere (Table 5). Furthermore, significant differences were recorded between the two seasons in the nodule number and weight, but no significant differences were recorded in the nitrogen fixed and the amount of nitrogen derived from the atmosphere between the two seasons (Table 5). No significant differences were recorded in the nodule number, weight, nitrogen fixed, and the amount of nitrogen derived from the atmosphere in the interaction of genotype with strains and genotypes with season (Table 5). Significant differences were recorded in the nodule number, but no significant differences were recorded in the nodule weight, nitrogen fixed, and nitrogen derived from the atmosphere in the interaction of genotypes with strains with soil status with the season (Table 5). Higher significant differences were recorded among inoculated BGN genotypes TVSu-787, TVSu-475, and TVSu-1739 in the pot experiment on nodulation and nitrogen fixation (Table 6) and were also recorded in the field studies. There were no significant differences recorded among TVSu-787, TVSu-1739, TVSu-305, TVSu-1,606, and TVSu-378 in the number of nodules observed in the glasshouse. Furthermore, there were no significant differences recorded among TVSu-475, TVSu-506, and TVSu-1,698 in the number of nodules in the glasshouse, but they were significantly higher than TVSu-365 and TVSu-710, which are not significantly different in the number of nodules recorded (Table 6). There were also no significant differences recorded among TVSu-475, TVSu-1,606, TVSu-1,698, TVSu-365, and TVSu-710 in the amount of nitrogen derived from the atmosphere, but it was significantly higher than other inoculated BGN genotypes in the glasshouse (Table 6). Inoculation of nitrogen fixing bacteria in the glasshouse are not significantly different in the nodulation and nitrogen fixation recorded but are significantly higher than nitrogen fertilizer applied and uninoculated control. A significant difference was recorded among the strains inoculated in nodule number, nodule weight, and nitrogen fixed (Table 7). Moreover, significant differences were recorded in soil status, seasons, and some interactions in the glasshouse (Table 7). The soil analysis results after growing the BGN genotypes (Bambara groundnut) eventually revealed that inoculation with strains had a significant effect on the soil, even after uptake and harvesting of the legume with high nitrogen content, organic carbon, and other soil properties (Table 8). The result obtained showed that TVSu-1739 showed the greatest significant differences in the nodule numbers recorded in both locations and seasons compared to other Bambara groundnut genotypes (Figure 3). Furthermore, TVSu-475 showed a greater significant difference than TVSu-1,698, which is significantly higher than other Bambara groundnut genotypes in the number of nodules recorded in both locations and seasons (Figure 3). No significant differences were recorded between TVSu-710 and TVSu-1,606 in the number of nodules recorded in both locations and seasons (Figure 3). The least number of nodules was recorded in TVSu-506 in both locations and seasons (Figure 3). The highest weight of nodules was recorded in TVSu-475, which was significantly higher than TVSu-1,698, significantly higher than TVSu-787 and TVSu-1739, and significantly higher than other Bambara groundnut genotypes in the weight of nodules recorded in both locations and seasons (Figure 2). Different variability was recorded among the Bambara groundnut genotypes in the amount of Nitrogen fixed in both locations and seasons (Figure 4). The highest nitrogen fixed values were recorded in TVSu-787, which was not significantly different from TVSu-1,698 but was significantly higher than other Bambara groundnut genotypes in the amount of nitrogen fixed in both locations and seasons (Figure 4).

**Table 4:** Nodulation and nitrogen fixation of BGN genotypes inoculated with nitrogen-fixation bacteria strains in Ibadan and Ikenne in both seasons.

Genotype	Strains	Nodule number	Nodule weight (g)	Nitrogen fixed (kg/ha)
TVSu-305	FA3	4.70 ± 5.40	1.04 ± 1.93	68.22 ± 17.81
	IRJ2180A	4.62 ± 7.24	1.24 ± 2.56	73.57 ± 13.01
	N	2.32 ± 4.22	0.50 ± 1.05	40.91 ± 5.80
	RACA6	5.92 ± 6.76	0.85 ± 1.70	72.52 ± 13.83
	USDA110	5.64 ± 7.49	0.99 ± 1.59	62.63 ± 11.87
	control	4.06 ± 6.38	0.73 ± 1.41	42.29 ± 4.14
TVSu-365	FA3	10.41 ± 9.3	1.26 ± 1.48	70.86 ± 12.29
	IRJ2180A	6.83 ± 5.81	1.13 ± 1.58	73.11 ± 9.63
	N	5.86 ± 5.55	1.89 ± 2.20	43.64 ± 6.47
	RACA6	4.17 ± 4.51	1.14 ± 1.75	81.10 ± 9.52
	USDA110	5.48 ± 5.55	1.35 ± 1.75	73.26 ± 14.22
	control	8.15 ± 6.16	1.85 ± 2.39	42.29 ± 4.14
TVSu-378	FA3	8.26 ± 5.85	1.08 ± 1.68	71.09 ± 9.89
	IRJ2180A	5.99 ± 6.56	0.69 ± 1.39	65.31 ± 15.92
	N	5.09 ± 4.72	0.82 ± 1.16	37.07 ± 8.14
	RACA6	6.69 ± 6.53	2.05 ± 2.99	72.64 ± 8.49
	USDA110	10.96 ± 6.9	1.73 ± 1.83	73.42 ± 10.96
	control	6.73 ± 6.88	1.53 ± 2.52	41.27 ± 4.96
TVSu-506	FA3	4.52 ± 7.41	0.63 ± 1.18	67.14 ± 5.39
	IRJ2180A	2.37 ± 5.52	0.69 ± 1.61	66.37 ± 11.70
	N	1.80 ± 3.08	0.49 ± 1.14	38.89 ± 4.90
	RACA6	3.13 ± 5.95	0.68 ± 1.43	73.86 ± 13.59
	USDA110	2.83 ± 4.48	0.70 ± 1.53	68.10 ± 6.45
	control	4.06 ± 4.39	0.91 ± 1.31	41.82 ± 4.94
TVSu-710	FA3	2.15 ± 2.71	0.33 ± 0.71	76.51 ± 12.83
	IRJ2180A	5.51 ± 5.56	0.81 ± 1.29	76.84 ± 9.96
	N	2.31 ± 2.72	0.94 ± 2.38	40.45 ± 10.57
	RACA6	4.29 ± 7.78	0.43 ± 0.75	72.19 ± 17.97
	USDA110	6.11 ± 8.12	0.78 ± 1.10	75.54 ± 16.80
	control	4.65 ± 5.57	0.61 ± 0.92	41.82 ± 8.94
TVSu-787	FA3	8.42 ± 9.22	1.60 ± 2.23	73.06 ± 9.94
	IRJ2180A	7.30 ± 4.96	1.59 ± 2.05	71.21 ± 12.53
	N	5.38 ± 4.93	0.96 ± 1.52	46.74 ± 11.13
	RACA6	11.30 ± 12.6	2.27 ± 2.88	76.88 ± 5.83
	USDA110	6.95 ± 5.23	1.27 ± 1.59	76.30 ± 9.23
	control	7.28 ± 6.69	1.67 ± 2.28	50.57 ± 18.03
TVSu-1,698	FA3	10.76 ± 10.8	1.57 ± 1.81	64.87 ± 22.51
	IRJ2180A	8.49 **±** 9.89	1.16 ± 1.56	79.69 ± 10.21
	N	4.59 ± 7.18	0.84 ± 1.49	48.92 ± 13.55
	RACA6	7.80 ± 6.00	2.16 ± 2.62	78.32 ± 13.38
	USDA110	7.21 ± 5.64	1.36 ± 1.61	75.92 ± 13.23
	control	11.7 ± 12.7	3.08 ± 4.38	45.08 ± 12.30
TVSu-1739	FA3	12.9 ± 12.0	1.29 ± 1.46	75.08 ± 10.79
	IRJ2180A	14.6 ± 14.5	1.61 ± 2.29	71.01 ± 17.69
	N	10.1 ± 10.4	0.99 ± 1.29	40.96 ± 6.03
	RACA6	14.5 ± 10.9	1.84 ± 1.92	70.86 ± 15.33
	USDA110	13.4 ± 5.96	1.66 ± 1.64	74.43 ± 12.56
	control	9.49 ± 7.95	1.18 ± 1.19	42.59 ± 5.60
TVSu-475	FA3	10.6 ± 12.9	1.89 ± 2.66	61.09 ± 12.43
	IRJ2180A	12.3 ± 9.65	1.98 ± 2.49	66.99 ± 12.87
	N	13.6 ± 9.60	2.94 ± 3.38	43.77 ± 5.93
	RACA6	10.2 ± 5.43	2.49 ± 2.69	77.33 ± 11.13
	USDA110	6.79 ± 8.88	2.01 ± 4.43	77.37 ± 4.32
	control	11.7 ± 9.68	2.24 ± 2.35	44.54 ± 5.03
TVSu-1,606	FA3	3.36 ± 4.48	0.53 ± 1.20	75.08 ± 9.07
	IRJ2180A	6.98 ± 10.7	0.77 ± 1.55	77.87 ± 12.02
	N	2.33 ± 4.52	0.25 ± 0.83	40.56 ± 3.59
	RACA6	5.17 ± 7.23	0.81 ± 1.52	82.99 ± 4.99
	USDA110	3.88 ± 5.62	0.74 ± 1.53	73.09 ± 18.27
	control	2.92 ± 3.30	0.38 ± 0.97	40.32 ± 5.51
CV (%)		106.6	117.6	17.1

Mean ± Standard error.

**Table 5:** Effect of nitrogen-fixation bacteria strains on nodulation and nitrogen fixation of BGN genotypes under glasshouse conditions in both seasons.

Source	DF	Nodule number	Nodule weight (g)	Nitrogen fixed (kg/ha)	% Ndfa
Genotypes	9	72.26*	0.41^ns^	25.1^ns^	1709.2*
Strains	5	975.45**	3.05**	0.55*	532.2^ns^
Soil status	1	376.65*	0.70 ns	28.7**	303.8^ns^
Season	1	3249.73**	146.45**	55.6^ns^	520.1^ns^
Genotypes*Strains	45	28.40^ns^	0.09 ns	24.6^ns^	532.3^ns^
Genotypes*Soil Status	9	27.73^ns^	0.71*	26.6^ns^	361.2^ns^
Genotypes*season	9	22.12^ns^	0.30 ns	36.0^ns^	491.3^ns^
Strains*Soil Status	5	67.50*	1.33**	53.5^ns^	540.9^ns^
Strains*season	4	365.46**	3.51**	23.5^ns^	1044.6^ns^
Genotypes*Stra*Soil*season	131	35.91*	0.19 ns	30.3^ns^	801.0^ns^
Rep	2	433.08**	7.06**	26.9^ns^	3608.1*

**p < 0.01 Significant, *p < 0.05 highly significant, ns-not significant.

**Table 6:** Effect of nitrogen-fixation bacteria strains on nodulation and % nitrogen derived from the atmosphere of BGN genotypes under glasshouse conditions in both seasons.

Genotypes	Nodule number	% Ndfa
TVSu-787	12.02^a^	31.03^b^
TVSu1739	11.35^ab^	33.48^b^
TVSu-305	10.96^abc^	36.73^b^
TVSu1606	10.62^abcd^	38.59^ab^
TVSu-378	10.08^abcd^	35.79^b^
TVSu-475	9.76^bcd^	47.93^a^
TVSu-506	9.58^bcd^	32.52^b^
TVSu1698	9.35^bcd^	40.59^ab^
TVSu-365	9.11^dc^	37.36^ab^
TVSu-710	8.64^d^	39.89^ab^

Means with the same letters are not significantly different at a 5% level of probability using the (DMRT).

**Table 7:** Effect of *Bradyrhizobium japonicum* strains on nodulation and Nitrogen fixation of BGN genotypes under glasshouse conditions in both seasons.

Strains	Nodule number	Nodule weight (g)	Nitrogen fixed (kg/ha)
FA3	11.10^a^	0.62^b^	29.8^a^
USDA110	11.12^a^	0.57^b^	29.3^a^
IRJ2180A	12.34^a^	0.66^b^	29.6^a^
RACA6	11.36^a^	0.57^b^	28.4^a^
N	10.75^a^	1.00^a^	28.2^a^
CONTROL	4.55^b^	0.22^c^	15.1^b^

Means with the same letters are not significantly different at a 5% level of probability using the (DMRT).

**Table 8:** Soil analysis after growing of nodulating legumes in locations.

Soil properties	Ikenne 2019	Ikenne 2020	Ibadan 2019	Ibadan 2020
pH (H_2_O) 1:1	4.86	4.85	6.65	6.61
N (%)	0.09	0.09	0.08	0.07
OC (%)	0.44	0.49	0.45	0.59
Bray P (ppm)	23.17	24.22	12.69	18.02
Sand (%)	76.00	78.00	82.00	83.00
Clay (%)	18.00	16.00	10.00	11.00
Silt (%)	6.00	6.00	8.00	6.00
Ca (Cmol/kg)	1.32	1.42	0.85	0.64
Mg (Cmol/kg)	0.42	0.38	0.04	0.04
K (Cmol/kg)	0.27	0.37	0.16	0.04
Na (Cmol/kg)	0.07	0.07	0.07	0.078
Zn (ppm)	1.19	1.62	1.38	1.64
Cu (ppm)	2.26	0.95	0.22	0.385
Mn (ppm)	15.29	11.88	136.74	133.39
Fe (ppm)	28.38	20.92	133.33	128.67

## 4. Discussion

Inoculation of BGN accessions *with B. japonicum* strains showed diverse responses in nodulation and nitrogen fixation in relation to geographical locations and seasons. Significant differences were observed among BGN genotypes and interactions of BGN genotypes and strains in nodulation and nitrogen fixation in Ibadan and Ikenne in 2019 and 2020. These findings are in accordance with Htwe et al. (2018), who reported that inoculation with *B. japonicum* singly enhanced nodulation and nitrogen fixation of soybean. Furthermore, significant differences were recorded among strains inoculated to BGN genotypes during nodulation in Ibadan 2019, but there were no significant differences recorded among the bacterial strains during nodulation in Ibadan in 2020 and Ikenne in 2019 and 2020, respectively, which is in agreement with the findings by Egamberdieva et al. (2018), who reported that the lowest number of nodules was observed on soybean roots grown under a low Phosphorus supply, regardless of the Nitrogen supply level. The variability recorded in the nodule number, weight, and nitrogen fixation in BGN genotypes was a result of the different genetic compositions of BGN genotypes and in response to the nitrogen-fixation bacteria strains. Notably, diverse with *B. japonicum* strains improved the nodulation (nodule number and nodule weight) of BGN genotypes in both locations and years, though no significant differences were recorded among the bacteria strains inoculation, which is in accordance with the findings of Leggett et al. (2017), who reported that inoculation with *B. japonicum* enhanced the nodulation of soybeans in the United States and Argentina in different years. Conversely, inoculation with bacteria strains also enhanced the nitrogen fixation of BGN genotypes in Ibadan and Ikenne in both years, which is in agreement with a study by Zimmer et al. (2016), which reported that inoculation with *B. japonicum* enhanced the nitrogen content of soybean. Mbah and Dakora (2018) observed that *B. japonicum* inoculation enhanced the nodulation and nitrogen fixation of Kersting groundnut and Bambara groundnut. An increase of over 20% was recorded among accessions in Ibadan and Ikenne in 2020 compared to 2019 in nodule number, nodule weight, and nitrogen fixed. Inoculation with FA3 and RACA6 strains significantly enhanced nodule number and nitrogen fixation, respectively, in Ibadan and Ikenne in both years compared to other bacterial strain inoculation and uninoculated controls (Table 5). Moreover, inoculation with FA3 strains enhanced TVSu-1739 more than other inoculated BGN genotypes in nodules number and weight and nitrogen fixed both in Ibadan and Ikenne in both years, but there were no significant differences recorded among TVSu-1,698, TVSu-475, and TVSu-365 (Table 5). In the glasshouse, bacteria strain inoculation also enhanced the nodulation and nitrogen derived from the atmosphere of BGN genotypes compared to the uninoculated control in both sterile and non-sterile soil, which is related to a study conducted by Adegboyega et al. (2021), which revealed that the results recorded in nitrogen fixation and nitrogen derived from the atmosphere in the shoot and root of wing bean genotypes. Inoculation with *B. japonicum* strains significantly enhanced nodule number and nitrogen derived from the atmosphere in TVSu-787 and TVSu-475, respectively, compared to other inoculated BGN genotypes (Table 8). This study revealed the response of inoculated BGN genotypes in both locations and seasons and in the glasshouse. A greater response was recorded among genotypes in the nodule number and weight, nitrogen fixation, and nitrogen derived from the atmosphere in relation to bacteria strain inoculation. Inoculation with nitrogen-fixation bacteria strains could help improve the nitrogen-fixation potential of genotypes, thereby improving the productivity of the BGN. However, some bacterial strains did promote the nitrogen fixation of BGN genotypes in this study, which was due to the colonization of the bacteria on the host plant and the nitrogen-fixation efficiency of the bacteria strains. BGN is usually nodulated by *Bradyrhizobium* spp. The significant impact of this bacteria strain on nodulation and nitrogen fixation often results in the promotion of the growth traits and flowering stages of BGN genotypes. The use of bacterial strains to inoculate BGN genotypes improved the nodule number, weight, and nitrogen fixed compared to the uninoculated control, which revealed the potentiality of the bacteria strain over chemical fertilizers. However, the bacteria strain is cheaper and causes no contamination after use compared to chemical fertilizers. The variation recorded among the Bambara groundnut genotype and bacteria strains inoculated in the nodule number, weight, nitrogen fixed, and amount of nitrogen derived from the atmosphere was due to the vast potential of the Bambara groundnut genotypes and the interaction of the various bacteria strains used in the study.

## 5. Conclusion

The inoculation of BGN genotypes with nitrogen-fixation bacteria strains enhanced the nodulation potential and nitrogen fixation of BGN genotypes. When legume seeds are coated with nitrogen-fixation bacteria before planting, the bacteria develop a mutual relationship with the root of the legume and develop inside the nodule structure by converting the atmospheric nitrogen to a form utilizable to BGN genotypes for optimum fixation and, thus, improve the yield output of the legumes for food sustainability and reduce the problem of food shortage. In this study, the FA3 and RACA6 strains significantly enhanced the nodulation potential and nitrogen fixation of BGN genotypes compared to other bacteria strains used in the study and uninoculated controls. Therefore, the use of bacteria strains to replace the use of chemical fertilizers can help to improve the nodulation potential and nitrogen fixation of BGN genotypes. Furthermore, the use of bacterial strains has no residual effect on the soil after planting, unlike chemical fertilizers. Limitations include the unavailability of the bacteria strains in most research stations and less accessibility to the end users, i.e., rural farmers.

## Data availability statement

The original contributions presented in the study are included in the article/supplementary material, further inquiries can be directed to the corresponding authors.

## Author contributions

TB: conceptualization, methodology, and writing original draft preparation. OB and OO: supervision and editing. MA: project administration and fund acquisition. AE and ET: analysis of the data. All authors discussed the results and contributed to the final manuscripts.
